# Phenotype-Driven Plasma Biobanking Strategies and Methods

**DOI:** 10.3390/jpm5020140

**Published:** 2015-05-14

**Authors:** Erica A. Bowton, Sarah P. Collier, Xiaoming Wang, Cara B. Sutcliffe, Sara L. Van Driest, Lindsay J. Couch, Miguel Herrera, Rebecca N. Jerome, Robbert J. C. Slebos, William E. Alborn, Daniel C. Liebler, Candace D. McNaughton, Ray L. Mernaugh, Quinn S. Wells, Nancy J. Brown, Dan M. Roden, Jill M. Pulley

**Affiliations:** 1 Institute for Clinical and Translational Research, Vanderbilt University, Nashville, TN 37203, USA; E-Mails: sarah.p.collier@vanderbilt.edu (S.P.C.); sunny.wang@vanderbilt.edu (X.W.); rebecca.jerome@vanderbilt.edu (R.N.J.); jill.pulley@vanderbilt.edu (J.M.P.); 2 Vanderbilt Technologies for Advanced Genomics Core Facility, Vanderbilt University, Nashville, TN 37232, USA; E-Mails: cara.b.sutcliffe@vanderbilt.edu (C.B.S.); lindsayjcouch@gmail.com (L.J.C.); miguel.herrera@vanderbilt.edu (M.H.); 3 Departments of Pediatrics and Medicine, Vanderbilt University Medical Center, Nashville, TN 37232, USA; E-Mail: sara.van.driest@vanderbilt.edu; 4 Jim Ayers Institute for Precancer Detection and Diagnosis, Vanderbilt University, Nashville, TN 37232, USA; E-Mails: r.slebos@vanderbilt.edu (R.J.C.S.); william.alborn@vanderbilt.edu (W.E.A.); daniel.liebler@vanderbilt.edu (D.C.L.); 5 Department of Biochemistry, Vanderbilt University Medical Center, Nashville, TN 37232, USA; E-Mail: r.mernaugh@vanderbilt.edu; 6 Department of Emergency Medicine, Vanderbilt University, Nashville, TN 37232, USA; E-Mail: candace.mcnaughton@vanderbilt.edu; 7 Departments of Medicine and Pharmacology, Vanderbilt University Medical Center, Nashville, TN 37232, USA; E-Mails: quinn.s.wells@vanderbilt.edu (Q.S.W.); nancy.j.brown@vanderbilt.edu (N.J.B.); dan.roden@vanderbilt.edu (D.M.R.); 8 Vanderbilt Translational and Clinical Cardiovascular (VTRACC) Research Group, Vanderbilt University Medical Center, Nashville, TN 37232, USA

**Keywords:** Keywords: biobanking, plasma, proteomics, BioVU, biorepository

## Abstract

Biobank development and integration with clinical data from electronic medical record (EMR) databases have enabled recent strides in genomic research and personalized medicine. BioVU, Vanderbilt’s DNA biorepository linked to de-identified clinical EMRs, has proven fruitful in its capacity to extensively appeal to numerous areas of biomedical and clinical research, supporting the discovery of genotype-phenotype interactions. Expanding on experiences in BioVU creation and development, we have recently embarked on a parallel effort to collect plasma in addition to DNA from blood specimens leftover after routine clinical testing at Vanderbilt. This initiative offers expanded utility of BioVU by combining proteomic and metabolomic approaches with genomics and/or clinical outcomes, widening the breadth for potential research and subsequent future impact on clinical care. Here, we describe the considerations and components involved in implementing a plasma biobank program from a feasibility assessment through pilot sample collection.

## 1. Introduction

### 1.1. Perspectives from DNA Banking

The BioVU DNA biobank provides a substantial platform for discovery in the area of personalized medicine. BioVU efforts capitalize on the availability of patient blood biospecimens remaining after routine clinical care and the ability to link these samples to the patient’s de-identified electronic medical record (EMR). After three years of design and engagement of university, operational, ethical, legal and community oversight, BioVU initiated accrual of adult DNA samples in 2007, and pediatric sample collection began in March of 2010. BioVU accumulation has operated under an opt-out, “all comers” approach, whereby any remaining blood biospecimens are collected from the Vanderbilt University Medical Center unless the patient decides to opt-out. There are no phenotypic exclusions, and BioVU banks one tube of DNA per individual. BioVU accumulation ranges between 1,200–2,400 unique adult and 160–320 unique pediatric DNA samples per month, and on average, 11,165 duplicate samples are discarded per month. A critical component to the success of BioVU is the linking of DNA samples to longitudinal medical records in the de-identified EMR database, known as the Synthetic Derivative (SD). These methods and results have been previously published [1,2,3,4,5,6,7].

**Figure 1:**
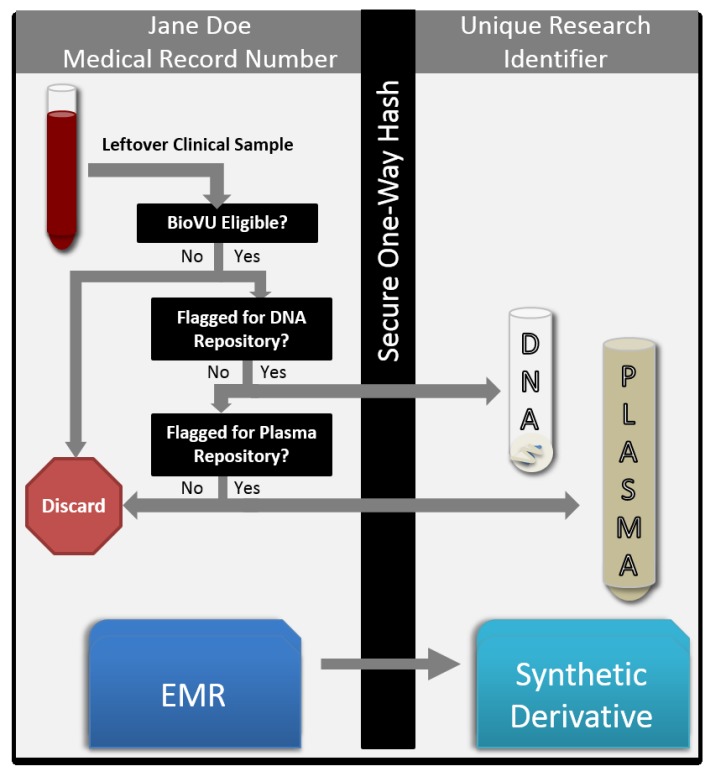
BioVU plasma biobanking is supported by existing DNA and EMR de-identification infrastructure. Leftover blood samples are scanned and queried against a database for BioVU DNA biobanking eligibility. Should DNA be already banked, the sample is considered for phenotypic plasma extraction. Both DNA and plasma are linked to their EMR through scrubbing by a secure one-way hash algorithm. Samples and records are linked via a de-identified research identifier.

DNA isolation and banking from eligible peripheral blood biospecimens is a well-established, highly-automated process carried out at a core laboratory facility within the Vanderbilt Medical Center. During the process of DNA extraction from whole blood, ancillary blood products, like serum and plasma, are discarded; thus the addition of plasma collection presents an opportunity to maximize the utility of the leftover blood specimens (Figure 1).

Similar to the DNA biobank, candidate proteomic biomarker discovery from human plasma holds incredible clinical potential, as well as significant challenges. Plasma biobanking demonstrates broad utility [8,9,10,11,12], widening the breadth of basic biomedical and clinical research. The dynamic range of proteins within plasma is known to exceed 10^10^, and many potential biomarkers are likely present at lower protein abundances [9]. Plasma may contain some residual and potentially detectable combination of all differentiated sub-proteomes of the body, affording the opportunity to gain information regarding all tissues and almost any disease state. Given the potential utility of plasma samples coupled with EMR data, as well as DNA, we have initiated phenotype-focused plasma collection into our institutional biobank. Herein, we describe our strategies and lessons learned during the design, pilot and moving towards the implementation of plasma biobanking to the BioVU resource.

### 1.2. Considerations for Plasma Collection

An important consideration relates to the dynamic nature of plasma biomarkers; whereas germline DNA typically does not change over time, plasma biomarkers are representative of health at the specific point in time from which blood was drawn, and thus, the time point and conditions of collection (within the context of health and disease) need to be known and integrated into subject selection protocols *a priori*. In conceptualizing the expansion of BioVU biobanking to include plasma, we considered the immediate utility of the large pool of duplicate samples currently discarded if DNA is already banked in an adequate quantity for that individual (Table 1). This strategy allows plasma banking to commence without interference with our established DNA program. Existing infrastructure for de-identification of samples and linkage to clinical records within the SD can be readily applied with minimal additional effort (Figure 1).

**Table 1:** Volume of leftover blood samples per month. Leftover blood samples are scanned daily to determine BioVU DNA biobanking eligibility. Samples from subjects who already have DNA samples banked (duplicate samples) are eligible for plasma biobanking. The number of blood samples are represented as the mean, median and standard deviation per month since 2006 through 2014.

	Mean	Standard Deviation	Median
Total scanned samples	35,519	5,527	36,409
Total duplicate samples scanned	11,165	2,063	11,437

Despite the applicability of the existing infrastructure to plasma banking, there were several key modifications to consider. The first was to determine the scale of the plasma bank. In large-scale DNA biobanking, collection of one sample for every individual eligible for biobank inclusion requires sample processing and storage of an average of 1,500 samples each month. The accrual rate for all potential serial plasma samples is over 11,000 monthly, nearly 10-fold more. Furthermore, plasma processing techniques require additional manual and automated processing outside of the protocols for the isolation of DNA from whole blood and storage of plasma samples at −80 °C to preserve protein stability (rather than −20 °C, adequate for DNA). Thus, a plasma collection program accruing all available samples would require a concomitant ~10-fold increase in resources (personnel, equipment, storage space, *etc.*). Given practical constraints, the initial BioVU plasma collection has been implemented using a targeted approach in which samples are collected by phenotype. Phenotype-specific accumulation, in contrast to the “all comers” approach employed for DNA biobanking, increases the utility for a given investigator, as plasma is intentionally banked. As described in greater detail below, this phenotype-driven selection adds a layer of complexity to the technical processes of selecting eligible patients; patients must meet certain phenotype criteria in addition to the standard eligibility criteria, and patients may meet criteria for more than one phenotype. Serial samples from the same individual may then be collected over time, enabling the comparison of biomarker levels across the course of a disease and/or treatment; this serial collection is another difference from DNA collection, in which germline DNA extracted from blood does not generally change, and thus, a single sample obtained at any point in time is sufficient.

As the program hinges on the use of specimens left over after clinical testing, the blood is not immediately processed, but is stored at 4 °C for approximately three days. Whereas DNA remains relatively stable under these conditions, plasma proteins and metabolites can be subject to degradation or other alterations; the processing and storage conditions of the specimens may differentially affect the performance of proteomic assays for specific proteins. While some studies have shown few significant changes in peptide and protein identification in samples stored at room temperature or after undergoing multiple freeze-thaw cycles, the studies are limited by the breadth of proteins measured and the type of detection technique [13]. This is an important consideration for the use of plasma within a model such as BioVU, and a further rationale for employing an investigator-driven collection model as the feasibility of target protein detection should be evaluated on an individual study basis. Of the three pilot studies conducted, described further below, all demonstrated the feasibility of protein detection and little difference between the quality of data in BioVU specimens compared to fresh specimens. While we focus here on the development of the infrastructure to enable use of plasma for a wide range of studies, we acknowledge the limitations of our model and note that not all proteomic studies are feasible using such an approach.

## 2. Results and Discussion

### 2.1. Input and Approvals

From its conception, BioVU has relied on the expertise of its advisory boards, including the Community Advisory Board, the Ethics Advisory Board, the Medical Center Ethics Committee and the Operational Oversight Board [4,6]. To successfully expand the program, early involvement of these boards was critical to ensure that technical, ethical and operational considerations were addressed prior to implementation. Transparency within the community is one of BioVU’s Principles of Operation, and thus, favorable support from the community to move forward with this expansion was sought. In discussions with the Community Advisory Board, it was suggested that the concept of “plasma” was not unlike that of “blood” or DNA extracted from blood and that attempts to differentiate the concepts with explanations might be counterproductive and confusing. Thus, in the updating of patient education materials and the BioVU public website (https://victr.vanderbilt.edu/pub/biovu), technical distinctions between plasma and extracted DNA were not attempted; content was updated to reflect the expanded use of “leftover blood”, and new examples of potential research projects that might be supported by either blood component were described. Operationally, the same approvals established for DNA banking were obtained to begin collecting plasma, including approval and support of all advisory boards and the Vanderbilt IRB (which oversees the BioVU program on an annual, continuing review cycle).

### 2.2. Pilot Plasma Project

To confirm interest in an ancillary plasma-based resource, potential researchers were contacted to gauge overall interest and to develop a list of use cases that could be piloted. We identified three primary goals for the pilot phase: (1) provide initial data describing the validity of the proteomic analysis of specimens processed after three days at room temperature; (2) demonstrate the feasibility of phenotype-driven selection of eligible subjects for inclusion; and (3) operationalize the isolation and processing of plasma from leftover clinical blood samples. Three plasma projects were selected to pilot various uses of plasma banking and clinical interests: (1) screening of antibody production in response to the therapy; (2) examination of plasma drug levels and outcomes; and (3) biomarker discovery. Pseudocode algorithms were developed and deployed in the SD, incorporating combinations of ICD-9 and/or CPT codes, medication exposures and clinical lab tests. Sufficient numbers were obtained for each of the three pilot projects, and manual chart review for confirmatory analysis rendered final iterations of the phenotypes for active sample accumulation via bioinformatic flagging, as discussed in greater detail below. Lastly, samples of plasma were assessed by each team for detectable target molecules via standard methods, such as enzyme-linked immunosorbent assay (ELISA) and mass spectrometry, to validate downstream approaches.

### 2.3. Phenotype-Driven Selection of Subjects

**Figure 2:**
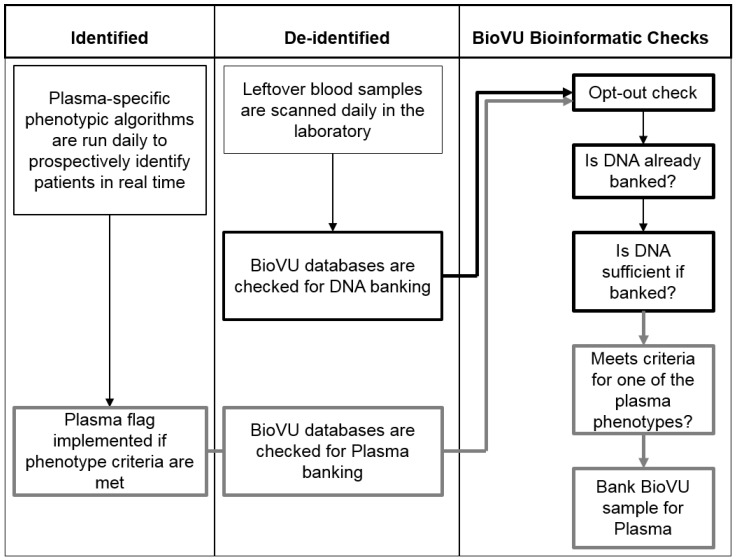
Schema of BioVU plasma bioinformatics processes and procedures. BioVU plasma collection requires the integration of three main data infrastructures. Daily plasma phenotypes are run against an identified database to identify eligible subjects who meet pre-defined clinical criteria. This information is then incorporated into the de-identified database, and only those subjects who already have a DNA sample banked are flagged for plasma collection.

Existing BioVU bioinformatics processes and procedures were leveraged to support plasma biobanking expansion, including a process of sample scanning at intake to determine sample inclusion (Figure 2). For plasma, the integration of a separate, identified database became necessary to determine plasma sample eligibility based on detailed clinical phenotype criteria available at the time of sample availability. The SD database could not be used for this step, as the de-identification process of the SD includes date-shifting, uncoupling the dates of relevant phenotype data from the actual date of sample collection. At the time of data analysis, both sample collection date(s) and all EMR data are shifted by the same number of days, but this process is not complete at the time of plasma sample selection. To operationalize the plasma inclusion protocol, pseudocode algorithms classifying plasma phenotypes are run over the entirety of the identified patient population daily, and eligible samples are indicated with a flag in the BioVU database. This approach does require significant manpower to incorporate and maintain the separate database with data pulls from multiple sources. Phenotype development, iteration testing for validation and writing code often requires several hours or even full days of effort. Moreover, maintenance of the identified and de-identified EMR databases, including clinical data integration and operational development, is the responsibility of a group of computer scientists within the Integrated Data Analyst Systems core facility.

**Figure 3:**
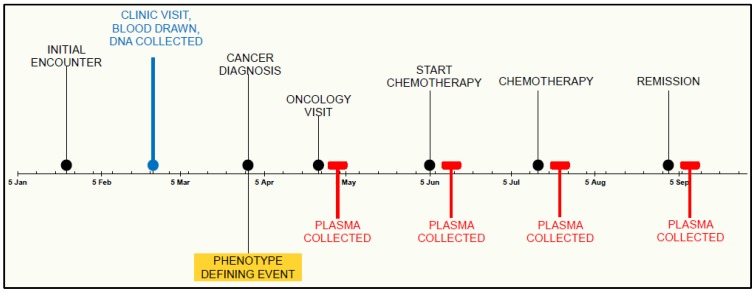
Example timeline of DNA and plasma parallel collection. BioVU DNA banking occurs in a defined moment in the medical timeline of a patient. DNA acquisition may be before, after or during diagnosis of a particular disease state. In contrast, phenotypic events trigger specific plasma collection, opening the possibility for observation throughout the varying phases of their clinical care.

In many instances, multiple blood tubes may exist for the same subject on the same day. For DNA collection, any tube beyond the one banked is considered a duplicate and is subsequently discarded. In phenotype-specific biobanking, one subject with multiple tubes could theoretically qualify for more than one plasma phenotype; to simplify, we implemented a one tube per subject per day cutoff for plasma banking. The frequency of accumulation can also be an issue for plasma phenotypes to support a successfully-powered study, and the rates of accumulation will vary. Thus, it was necessary to incorporate sophisticated temporal criteria into the sample selection and plasma flagging procedures to accommodate both subject qualification and sample qualification. In other words, the subject may meet phenotype criteria, but the timing of the plasma sample must also meet the pre-defined clinical requirements (e.g., a specific amount of time following a clinical event or serial sample collection with a minimum amount of time in between each sample collected) (Figure 3).

**Figure A1:**
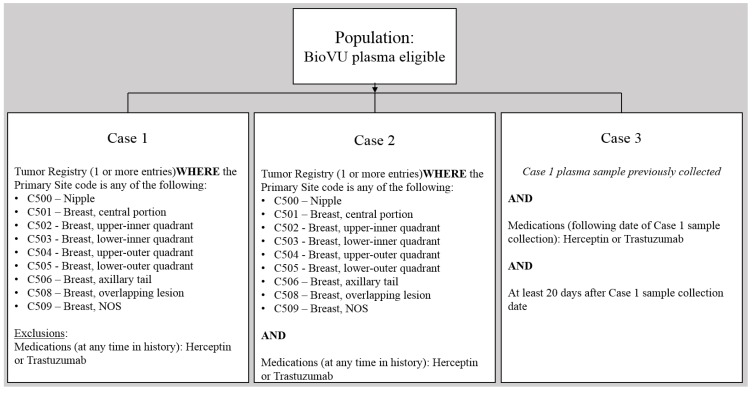
The sample plasma phenotype demonstrates clinical criteria used to define eligible samples in real time.

Algorithms for defining each phenotype are developed using a variety of potential strategies and data types, but are constrained by the requirement that the eligibility criteria be available in the EMR at the time of sample selection. For example, in our EMR system, all demographic, laboratory and vital sign data are available as formatted data elements immediately upon report and can be used to identify plasma samples for accrual. Previous diagnoses, defined based on ICD-9 codes, keywords in clinical documents or specific medication exposures, can also be used as inclusion or exclusion criteria. In contrast, discharge diagnoses for the current hospitalization are not typically entered until at or after discharge and would not be valid criteria. Each phenotype definition (potentially including both cases and controls or a spectrum of illness) must be developed and validated for each project; a well-established process in the context of BioVU research protocols and undertaken using an interactive, collaborative approach by the BioVU team and the investigator. A sample algorithm is shown in Figure A1.

As blood samples are scanned in the laboratory, they are checked against the BioVU database for DNA eligibility and plasma eligibility. Samples are only eligible for plasma collection if: (i) the subject did not opt-out; (ii) if DNA is already banked in BioVU; and (iii) they meet the inclusion criteria for an active plasma collection phenotype definition. Because the identified database is utilized only electronically to establish eligibility (similar to existing protocols to establish whether an individual patient has opted-out) prior to scanning of the leftover blood, all de-identification protocols are maintained. No human eyes ever see identified information on the samples.

### 2.4. Collection and Processing

Collection of plasma follows the processes and procedures in place for parallel DNA collection. Blood specimens collected as part of routine clinical care are stored at 4 °C until they are picked up by designated BioVU technicians 3–5 days post draw date. Technicians select blood specimens collected in EDTA-treated vacutainers during the daily pickup from the Central Pathology Department refrigerator. Upon arrival to the Vanderbilt Technologies for Advanced Genomics (VANTAGE) Core, the blood specimens are immediately processed. Specimens are placed in Tecan 16-position carriers and loaded on the deck of the Tecan Freedom EVO 200 used for scanning. The Tecan PosID (Positive Identification System) module scans the sample barcode to enable the selection of samples matching BioVU criteria and sorts the vacutainers based upon eligibility for DNA, as well as plasma collection. For those samples eligible for the plasma biobank, the blood is fractionated by centrifugation at 1,499× *g* for 15 min, and plasma fractions are inspected for sufficient volume. A minimum volume of 500 microliters is designated as the threshold in the workflow; thus, approximately 9% of eligible samples fail to be collected due to insufficient volume. The isolated plasma fractions are transferred and stored within 1.4-milliliter 2D barcode Matrix tubes (Thermo Scientific). Each 500-microliter plasma sample is scanned into the BioVU Laboratory Information Management System (LIMS) and stored at −80 °C until designated investigator pick-up date. Upon request from the investigator, a manifest is prepared from the LIMS, including 2D tube numbers with sample IDs, as well as the box position. Investigators pick up samples on a pre-arranged schedule, and samples are stored on dry ice during pick-up and transfer to the investigator.

### 2.5. Discussion

Two major considerations of plasma biobanking implementation are utility and cost. In contrast to the opt-out “all comers” approach to DNA banking, we designed plasma accumulation to be project focused and phenotype specific. This design reduces the resources necessary to process and store plasma and increases the potential for utility, particularly for protocols involving serial sample collection. By selectively collecting for investigator-driven projects, we have minimized the potential waste in banking of samples that might never be used. Limiting to one sample per eligible subject per day also eliminates duplication and reduces the workload required for processing and storage. However, BioVU leadership will closely monitor the demand for and success of projects using the plasma resource and will consider strategies for further expansion as our experience with archived plasma grows.

While plasma collection creates new opportunities for research and there is a clear benefit in collecting multiple samples from the same individual, there are also limitations in the capabilities of the BioVU design. Only leftover blood is used as a source of plasma; this blood is drawn for clinical purposes and not research purposes, thus investigators are limited to the plasma available at these time points. This limitation could potentially introduce design bias into BioVU studies. For example, a critical time point could be missed because of lack of blood sample availability at that time (e.g., following an adverse event or directly before beginning medication). A second issue is the potential for selection bias, as patients with chronic illnesses and/or medication exposures are more likely to be seen regularly and have their blood drawn for clinical purposes. However, approximately 60,000 patients are considered part of the Vanderbilt “medical home” (defined bioinformatically by three or more visits in two years to internal medicine providers for routine medical care) and are seen in clinics on a regular basis. It is crucial to consider the potential availability of leftover specimens for the specific disease or outcome being studied. In many instances, the study may not be feasible due to selection biases (e.g., depression, where blood sampling is not part of routine care); however, for diseases, such as cancer, where there exists protocoled monitoring of patients, the opportunities to collect leftover specimens are sufficient to initiate a study.

Another key issue worth close scrutiny in future work lies in the storage and handling conditions for plasma in this new component of the BioVU infrastructure. In general reflection of the plasma biobanking program described herein, we acknowledge potential limitations due to sample processing and storage delays from the time of draw, which may limit broad utility depending on the stability of the target analyte. Previous studies examining the stability of plasma and serum samples have suggested that certain proteins, mainly related to coagulation and complement pathways, are susceptible to proteolytic degradation or oxidation during and after blood collection and processing steps [14,15,16,17,18,19,20,21]; such proteins, such as cytokines, may not be amenable to study using our plasma resource. Many of the past studies use enrichment strategies that focus on the analysis of the low molecular weight proteome of serum using MALDI-TOF mass spectrometry; however, the overall global integrity of proteins, particularly in plasma, has not been previously described. Zimmerman *et al.* showed that analysis of plasma samples subjected to various collection conditions and freezing/thawing cycles followed by tryptic digestion and reversed-phase liquid chromatography mass spectrometry revealed minimal changes in proteins from plasma collected in EDTA tubes, with only modest degradation of those proteins previously shown to be highly susceptible to cleavage (e.g., fibrinogen, complement C3) [13]. These results showed non-specific degradation as a result of plasma storage conditions, and in general, most proteins remain unaffected in downstream analysis, similar to other investigations that revealed the stability of many proteins despite delayed processing or storage at less optimal temperatures [22,23]. Given the growing volume and diversity of literature describing the stability of specific proteins, metabolites and other characteristics of plasma samples under various collection and storage conditions, the validity of using plasma samples obtained via BioVU must be considered within the context of each associated research proposal. When extant literature is lacking, we would anticipate that interested researchers would elect to integrate a validation component into their protocols to support the utility of BioVU plasma samples for their objectives. We acknowledge that such validation work is essential, particularly in pilot studies on the individual project level, and will prove crucial to fully characterizing both the utility and the limitations of this resource. These data will have the added benefit of further increasing scientific knowledge of diverse plasma characteristics in different situations. 

## 3. Conclusions

In conclusion, we discuss our work to create a research resource providing a foundation that appeals broadly to a range of biomedical and clinical enterprises. Moreover, leveraging the existing DNA biobank linked to the SD, a new dimension is afforded by plasma collection and potential use. There are numerous advantages to linking protein-derived data with EMR-derived data. The de-identified EMR database allows for the study of rare disease or events that would otherwise be difficult to conduct and continues to be updated as patients return to receive clinical care, enabling longitudinal biomarker analysis. Moreover, the availability of DNA in ~200,000 subjects affords future opportunity to expand into the proteogenomic space to examine the genetic influences of proteomic measures within a cohort of subjects representing a host of diseases within the EMR system. One advantage to proteogenomic data integration is the support of research enterprises outside traditional genetics and favoring a genre of basic biomedical laboratory science, widening the appeal to the research community at large. With the addition of plasma availability, BioVU could support genomics, as well as epigenetics, proteomics, cytomics, metabolomics, interactomics and bioinformatics. The addition of this complexity;however, creates new practical and informatics challenges, including the integration and storage of data, as well as how the data will be shared for secondary use. The variety of analyses that can be performed using plasma, including protein, RNA and metabolite analyses, provides a substantial opportunity for a living snapshot of the whole body that cannot be gleaned entirely from peripheral DNA. Although there is considerable work to be done to fully calibrate the use of plasma with EMR data, as has been done for DNA biobanking, the new resource offers great potential for establishing how changes in proteomic profiles across time relate to health outcomes, including disease progression, response to treatments/medications and environmental exposures.

## Abbreviations

ICD: International Classification of Diseases
CPT: Current Procedural Terminology
